# Muscle-specific color stability in fresh beef from grain-finished *Bos indicus* cattle

**DOI:** 10.5713/ajas.18.0531

**Published:** 2018-11-28

**Authors:** Ana Paula A. A. Salim, Surendranath P. Suman, Anna C. V. C. S. Canto, Bruno R. C. Costa-Lima, Fernanda M. Viana, Maria Lucia G. Monteiro, Teofilo J. P. Silva, Carlos A. Conte-Junior

**Affiliations:** 1 Department of Animal and Food Sciences, University of Kentucky, Lexington, KY 40546, USA; 2 Chemistry Institute, Technology Center, Federal University of Rio de Janeiro, Rio de Janeiro, RJ 21941-909, Brazil; 3 Department of Food Technology, Federal Fluminense University, Niteroi, RJ, 24230-340, Brazil

**Keywords:** Beef, Meat Quality, Myoglobin, Nellore, Oxidation

## Abstract

**Objective:**

To investigate the color and oxidative stabilities of *longissimus lumborum* (LL) and *psoas major* (PM) muscles from grain-finished *Bos indicus* cattle in Brazil.

**Methods:**

The LL and PM muscles were obtained 24 h post-mortem from eight (n = 8) Nellore bull carcasses, fabricated into 1.5-cm steaks, aerobically packaged, and stored at 4°C for nine days. Steaks were analyzed for myoglobin concentration, pH, instrumental color, metmyoglobin reducing activity (MRA) and lipid oxidation.

**Results:**

The LL steaks exhibited greater (p<0.05) redness, color stability, and MRA than their PM counterparts on days 5 and 9. The LL and PM steaks demonstrated similar (p>0.05) lightness and yellowness on days 0, 5, and 9. On the other hand, PM steaks exhibited greater (p<0.05) myoglobin concentration, pH, and lipid oxidation than their LL counterparts.

**Conclusion:**

These results indicated that muscle source influenced the color and oxidative stabilities of beef from grain-finished *Bos indicus* animals. These results highlighted the necessity of muscle-specific strategies to improve the color stability of beef from grain-fed *Bos indicus* cattle.

## INTRODUCTION

Brazil has the largest commercial beef cattle herd, composed mainly of *Bos indicus* animals [1]. Nellore cattle are well-adapted to Brazilian climatic conditions and production systems, in which the animals are raised on pasture and finished on grain-based (corn) or forage-based (grass silage and sugar cane) diets during the 3 to 4 months preceding the harvest [2]. Beef color is the major quality attribute governing the consumers’ purchase decisions and any deviation from this color can lead to product rejection [3]. Myoglobin (Mb) is the protein responsible for meat color, and its redox state is influenced by extrinsic and intrinsic factors [4,5]. Muscles in a beef carcass demonstrate differences in biochemistry and composition [6–9], and muscle source is a major intrinsic factor influencing beef color stability [6]. *Longissimus lumborum* (LL) is composed of glycolytic fibers, whereas *psoas major* (PM) is composed mainly of oxidative fibers [7,10,11]; both muscles demonstrate differences in surface color, metmyoglobin reducing activity (MRA), and lipid oxidation [12–15]. Investigations in this aspect documented that beef color stability is a muscle-specific trait [6,11–15]. McKenna et al [6] evaluated the discoloration in 19 muscles from *Bos taurus* cattle and reported that muscles classified as color-stable (such as LL) exhibited greater a* values and color stability than muscles classified as color-labile (such as PM). Abraham et al [15] reported greater redness and MRA in LL than in PM steaks from *Bos taurus* cattle during 7 days of retail display. Moreover, Canto et al [16] documented greater surface redness, color stability, and MRA in LL than in PM steaks from grass-fed *Bos indicus* cattle during 9 days of refrigerated storage.

Breed also affects beef color stability [3]. Miguel et al [17] observed greater redness in *longissimus thoracis* from Nellore animals than their counterparts from crossbred (Nellore× Aberdeen Angus) animals. On the other hand, longissimus steaks from Nellore bulls demonstrated lower redness than the steaks from Caracu and Holstein Friesian (*Bos taurus*) bulls [18].

Among extrinsic factors, diet plays an important role in fresh meat color influencing the susceptibility of meat to oxidative deterioration [12,19,20]. Compared to grain-fed beef, fresh meat from pasture-fed cattle contains high levels of natural antioxidants [12,20], which in turn exert a protective effect against lipid oxidation and contribute to an increase in redness [12]. Nonetheless, investigations in this aspect have been demonstrating conflicting results. Tansawat et al [20] reported greater redness, lightness, and yellowness in longissimus muscles from grain-fed *Bos taurus* beef animals than those from pasture-fed counterparts. On contrary, lower redness was documented in longissimus muscle from grain-fed *Bos taurus* cattle compared to those from pasture-fed animals [12]. Although previous investigations studied the impact of muscle source on color stability of beef from *Bos taurus* [6,11,14,15], Chinese Luxi cattle [21], and grass-fed *Bos indicus* cattle [16], the influence of muscle source on beef color from grain-finished *Bos indicus* animals has not been investigated.

Therefore, the objective of the present study was to examine the color and oxidative stabilities of LL and PM muscles, from Nellore (*Bos indicus*) bulls finished in grain, during refrigerated storage under aerobic conditions.

## MATERIALS AND METHODS

### Experimental design and beef fabrication

Eight (n = 8) purebred Nellore (*Bos indicus*) bull carcasses (24 h post mortem) were used in this experiment. The bulls were raised under similar conditions in a farm on Uberaba (MG, Brazil). The bulls were pasture-fed until 15 to 21 months age. After this period, the animals were allocated in feedlots, and grain-fed (corn) for 3 months prior to harvest. The animals were harvested at 18 to 24 months age in a commercial facility located in Colatina (ES, Brazil). The carcasses had an average cold weight of 285.0 kg, and LL and PM muscles were excised from the right sides of the carcasses (24 h postmortem), individually vacuum packed, and shipped under refrigeration to the Universidade Federal Fluminense (Niteroi, RJ, Brazil).

All external fat was removed, and the LL and PM muscles were fabricated into ten 1.5-cm thick steaks. The steaks were individually packaged on polystyrene trays with soaker pads, over-wrapped with oxygen-permeable polyvinyl chloride film (0.014 mm thickness; 15,500 to 16,275 cm^3^/m^2^/24 h oxygen transmission rate at 23°C), and were assigned randomly for 0, 5, and 9 days at 4°C in darkness. On day 0, four steaks were assigned for analyses of Mb concentration, pH, instrumental color, MRA and lipid oxidation. The remaining six steaks were utilized for evaluation of instrumental color and biochemical attributes on days 5 and 9 (three steaks/d; 1 for color and 2 for biochemical analyses). All the analyses were performed in duplicate.

### Myoglobin concentration

Mb concentration was determined according to method described by Faustman and Phillips [22]. Five-gram muscle samples were homogenized with 45 mL ice-cold sodium phosphate buffer (40 mM, pH 6.8) and filtered using Whatman no. 1 paper, followed by an additional filtering through a 22-μm membrane filter. The absorbance of the filtrate at 525 nm (A_525_) was recorded using a UV-1800 spectrophotometer (Shimadzu Corporation, Kyoto, Japan). The Mb concentration was calculated using the following equation:

$$\begin{array}{l} \text{Myoglobin }(\text{mg}/\text{g muscle tissue})\\ =[\text{A}525/(7.6\;{\text{mM}}^{-1}\;{\text{cm}}^{-1}\times 1\;\text{cm})]\times (17,000/1,000)\times 10 \end{array}$$

Where: 7.6 mM^−1^ cm^−1^ = mM absorptivity coefficient of Mb at 525 nm; 1 cm = light path length of cuvette; 17,000 Da = average molecular weight of Mb; 10 = dilution factor.

### Meat pH

The pH was measured utilizing a portable pH meter (Hanna Instruments US Inc., Woonsocket, RI, USA, equipped with an insertion type probe [23].

### Instrumental color evaluation

Surface lightness (L*), redness (a*), and yellowness (b*) values were measured using a portable spectrophotometer CM-600D (Konica Minolta Sensing Inc., Osaka, Japan) equipped with illuminant A, 8 mm aperture, and 10° standard observer [24]. Color was measured at three random locations on the steak surfaces. Additionally, color stability was indirectly estimated through the ratio of reflectance at 630 nm and 580 nm (R630/580) according to AMSA [24].

### Metmyoglobin reducing activity

MRA was evaluated according to method of Sammel et al [25]. Two cubes (2.0 cm×2.0 cm×2.0 cm) were sliced from each steak and individually submerged in sodium nitrite solution (0.3%) for 20 min to induce metmyoglobin formation. The cubes were arranged with their initially light-exposed side facing up in order to ensure contact with the nitrite solution. The cubes were blotted dry, vacuum packed, and the reflectance values (from 400 to 700 nm) were recorded using a portable spectrophotometer CM-600D (Konica Minolta Sensing Inc., Japan). After incubation at 30°C for 2 h allowing for metmyoglobin reduction, the previously evaluated surfaces were rescanned.

Metmyoglobin formation on surface was calculated utilizing the absorption coefficient/scattering coefficient (K/S) ratios and formulas according to AMSA [24], and the MRA was estimated using the following equation:

$$\begin{array}{l} \text{MRA}=100\times [(\text{pre-incubation percentage of surface}\\ \text{metmyoglobin})-(\text{post-incubation percentage of surface}\\ \text{metmyoglobin})]/[(\text{pre-incubation percentage of}\\ \text{surface metmyoglobin})] \end{array}$$

### Lipid oxidation

Lipid oxidation was evaluated using the method described by Yin et al [26]. Samples (5 g) were homogenized with 22.5 mL of 11% trichloroacetic acid solution and filtered through Whatman no. 1 paper. One milliliter filtrate was mixed with 1 mL of aqueous solution of thiobarbituric acid (20 mM) and incubated at 25°C for 20 h. The absorbance values at 532 nm were measured utilizing a UV-1800 spectrophotometer (Shimadzu Corporation, Japan), and were presented as thiobarbituric acid reactive substances (TBARS).

### Statistical analysis

Eight (n = 8) beef carcasses were utilized in this study, and the experimental design was completely randomized. Data were analyzed using XLSTAT software (Version 2014.5.03, Addinsoft, Inc., Brooklyn, NY, USA). One-way analysis of variance (ANOVA) was used for analysis of Mb concentration. A two-way ANOVA was utilized for analysis of pH, instrumental color, MRA, and lipid oxidation to assess the effect of muscle source (LL and PM) and days of storage (0, 5, and 9). Tukey’s test was used to compare treatment means at 5% significance level (p<0.05).

## RESULTS AND DISCUSSION

### Myoglobin concentration

Mb concentration was greater (p<0.05) in PM (4.66±0.31) steaks than in their LL (3.97±0.12) counterparts. The observed variations in Mb concentration between LL and PM steaks could be attributed to the differences in muscle fiber characteristics [8]. Muscles composed of type I fiber, such as PM, exhibited greater Mb concentration than glycolytic muscles, such as LL, in Hanwoo cattle [7,27]. Moreover, a greater Mb concentration has been previously associated with lower color stability of PM than in LL steaks [13,16]. Mb contains heme iron, a prooxidant capable of accelerating lipid peroxidation, which in turn favors formation of metmyoglobin and concomitant discoloration in fresh meats [21]. In agreement with our results, previous investigations documented greater Mb concentration in PM steaks than in LL from grass-fed *Bos indicus* [16] and Hanwoo [7,27] cattle. In contrast, McKenna et al [6] documented lower Mb concentration in PM than in longissimus steaks.

### Meat pH

The PM steaks exhibited greater (p<0.05) pH values than LL counterparts throughout the storage (Table 1). The differences in pH between LL and PM could be attributed to the differences in muscle fiber composition [9,19,27]. Muscles composed predominantly of fiber type IIB such as LL [27] exhibit greater content of glycogen and glycolytic potential [8] than muscles composed of fiber I such as PM [27]. The glycolytic metabolism of type IIB fiber promotes the use of glucose as energy source, leading to the post-mortem accumulation of lactic acid and resulting in a decrease of muscle pH [9]. Similarly, Canto et al [16] reported greater pH values in PM than in LL steaks from pasture-raised *Bos indicus* cattle on days 3, 6, and 9 of refrigerated storage. Moreover, previous studies reported greater pH values in PM muscles than in LL counterparts from *Bos taurus* cattle [6,9,14,15]. In contrast, Hwang et al [27] reported greater pH values of *longissimus thoracis et lumborum* than in PM muscle from Hanwoo cattle on day 0. Wu et al [21] did not observe differences in pH values of LL and PM muscles from Chinese Luxi cattle during 15 days of storage.

Storage influenced (p<0.05) the pH of LL and PM steaks; pH increased in both muscles (LL and PM) on day 9. This could be related to the postmortem proteolysis and generation of basic metabolites, such as amines, both of which contribute to the increase in pH during retail display [28]. In agreement, Joseph et al [14] documented an increase of pH in both LL and PM muscles from *Bos taurus* cattle, mainly on day 9 of retail display. Wu et al [21] also documented a pH increase in LL and PM muscles during 15-day of retail display. In partial agreement, Canto et al [16] documented a pH increase in PM steaks from day 3 to 9 of storage. On the other hand, Jeong et al [7] did not report differences in the pH of muscles *longissimus thoracis et lumborum* and PM from Hanwoo cattle during 7 days of storage. Kim et al [11] reported negligible changes in the pH of LL and PM muscles during 7 days of retail display.

### Instrumental color

**Figure 1:**
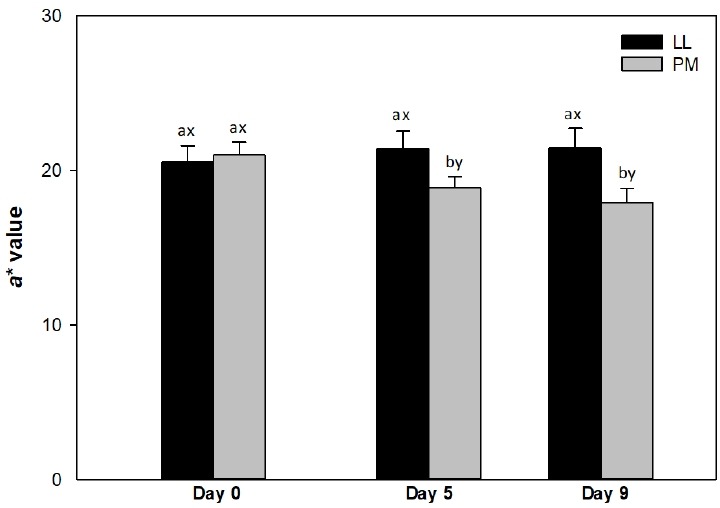
Surface redness (a* value) of *longissimus lumborum* (LL) and *psoas major* (PM) steaks from grain-fed Nellore (*Bos indicus*) bulls during aerobic storage at 4°C for 9 days. Standard error bars are indicated. ^a–b^ Means within a muscle without common superscripts are different (p<0.05). ^x–y^ Means within a day of storage without common superscripts are different (p<0.05).

**Figure 2:**
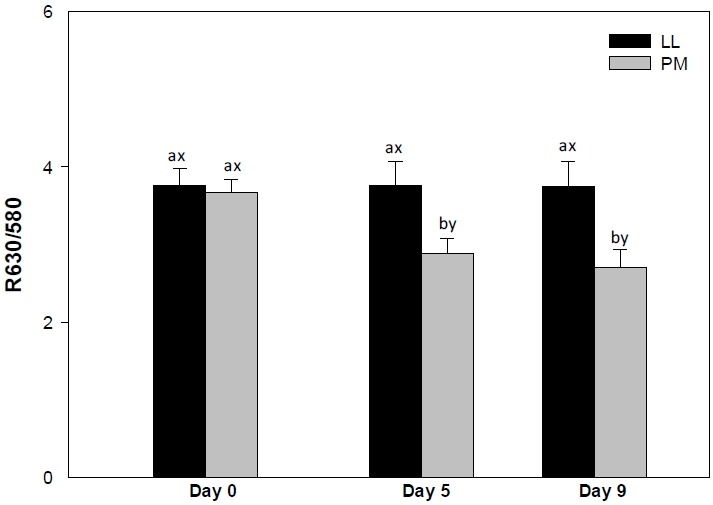
Surface color stability (R630/580) of *longissimus lumborum* (LL) and *psoas major* (PM) steaks from grain-fed Nellore (*Bos indicus*) bulls during aerobic storage at 4°C for 9 days. Standard error bars are indicated. ^a–b^ Means within a muscle without common superscripts are different (p<0.05). ^x–y^ Means within a day of storage without common superscripts are different (p<0.05).

Muscle source influenced (p<0.05) surface redness (a* values; Figure 1) and color stability (R630/580; Figure 2) of LL and PM steaks, whereas it did not impact (p>0.05) lightness (L* value; Table 1) and yellowness (b* value; Table 1).

#### Lightness (L* value)

The LL and PM steaks demonstrated similar (p>0.05) lightness (L* values) on days 0, 5, and 9 of storage. Nonetheless, the L* values were numerically greater in LL than in PM steaks. The observed difference in L* values between LL and PM could be attributed to the differences in muscle fiber composition [7,8,27]. Muscles composed of fiber type IIB, such as LL and *longissimus thoracis*, demonstrated greater lightness than muscles composed of fiber type I such as PM [27]. The glycolytic potential of fiber IIB favors the use of glycogen as energy source, leading to a rapid post-mortem pH decrease [9] and a reduction of water holding capacity of meat [8], which in turn influence the superficial light reflectance, affecting L* values [29].

In agreement, Joseph et al [14] and Kim et al [11] reported similar lightness in LL and PM muscles from *Bos taurus*, during storage. In partial agreement, Canto et al [16] reported similar lightness in LL and PM steaks from pasture-fed *Bos indicus* cattle on days 3 and 9 of storage. Wu et al [21] also reported similar L* values in LL and PM muscles of Chinese Luxi cattle from day 5 to 15 of retail display. In contrast, Hwang et al [27] and Jeong et al [7] reported greater L* values in *longissimus thoracis et lumborum* than in PM steaks from Hanwoo cattle.

While storage did not (p>0.05) affect the L* values of LL steaks, PM steaks exhibited a decreased in L* values (p<0.05) from day 5 of storage. McKenna et al [6] evaluated the discoloration in 19 muscles from *Bos taurus* cattle and reported that muscles classified as color-stable (such as LL) exhibited steady L* values throughout storage, whereas color-labile muscle (such as PM) demonstrated a decline in L* values during 5 days of retail-display. In partial agreement, Canto et al [16] reported that the storage did not affect L* values of LL steaks from grass-fed *Bos indicus* beef animals, whereas an increase in lightness was observed in PM on day 9. On contrary, Joseph et al [14] and Kim et al [11] documented no changes in L* values of LL and PM steaks during storage.

#### Redness (a* value)

The LL steaks exhibited greater (p<0.05) redness (a* values) than PM counterparts on days 5 and 9 (Figure 1), whereas both muscles demonstrated similar (p> 0.05) values on day 0. The differences in a* values observed between LL and PM steaks could be attributed to the differences on muscle fiber characteristics [7,27]. The PM is composed mainly of fiber type I, which exhibits greater Mb concentration [8,27] and oxidative metabolism [8] than the muscles composed of fiber type IIB, such as LL [27]. This in turn favors the oxidation of Mb to metmyoglobin [6,21] decreasing redness in PM than in LL steaks [15,21]. In addition, the decrease on a* values could be attributed to the low levels of antioxidant proteins in PM than in LL, which in turn favors Mb oxidation resulting in a rapid decline in redness of PM [14]. In agreement, a greater redness in LL than in PM steaks has been previously reported in *Bos taurus* [11,15] and Chinese Luxi cattle [21]) cattle during storage. Canto et al [16] documented greater surface redness in LL than in PM steaks from grass-fed *Bos indicus* cattle on days 0, 3, 6, and 9 of refrigerated storage. Our results differ with those of Canto et al [16] probably due to the variation in animal diet [30]. Pasture-based diets are rich in vitamin E, carotenes and polyphenols, all which act as free radical scavengers, and exerts a protective effect on muscle lipids [30]. This in turn, prevents the Mb oxidation and contributes to increase of redness [21]. Additionally, Joseph et al [14] documented greater a* values in LL steaks than in PM counterparts from *Bos taurus* cattle on days 5 and 9 of retail display. On contrary, Patten et al [9] reported greater a* values in PM than in LL muscles, from *Bos taurus* cattle on day 0 of storage.

The PM exhibited a decrease (p<0.05) in a* values from day 5, whereas the surface redness did not change (p>0.05) in LL steaks throughout the 9 days of refrigerated storage (Figure 1). The observed decline in a* values of PM steaks could be attributed to Mb oxidation and accumulation of metmyoglobin on surface during storage, resulting in a decrease of redness in PM [15]. Similarly, McKenna et al [6] reported that storage did not affect a* values of LL steaks, whereas PM exhibited a decrease pattern from day 0 to 5. In partial agreement, a decrease in a* values have been previously reported in both LL and PM muscles from *Bos taurus* [14,15] and grass-fed *Bos indicus* [16] cattle during storage.

#### Yellowness (b* value)

The LL and PM steaks exhibited similar (p>0.05) b* values on days 0, 5, and 9 (Table 1). Nevertheless, the LL steaks demonstrated numerically greater b* values than the PM counterparts throughout the storage. Supporting our results, similar b* values in both LL and PM muscles, from *Bos taurus* cattle, have been previously reported on day 0 of storage [9,14,27]. On the other hand, Canto et al [16] documented greater yellowness in LL than in PM steaks from grass-fed *Bos indicus* cattle during 9 days of refrigerated storage. In the present study, the animals were finished on grain-based diet, which has low availability of carotenoids than pasture-based ones. This in turn, may decrease the content of this yellow plant-based pigment in meat, and consequently influence the b* values [31]. McKenna et al [6] documented that color-stable muscles, such as LL, exhibited greater b* values than color-labile muscles, such as PM, throughout 5 days of retail-display. In addition, previous studies documented greater yellowness in LL than in PM muscles from *Bos taurus* [11] and Hanwoo [7] cattle during 7 days of storage.

Storage did not (p>0.05) affect the surface yellowness of LL and PM steaks. In support, Wu et al [21] reported similar b* values in LL and PM steaks from Chinese Luxi cattle during 5 days of storage. In partial agreement, Canto et al [16] reported that storage did not affect the b* values of PM steaks from grass-fed *Bos indicus* cattle, whereas LL exhibited a decrease in yellowness from day 3 to 9. In addition, Jeong et al [7] documented no difference on yellowness in LL steaks from Hanwoo cattle throughout the 7 days of storage. In contrast, Joseph et al [14] documented a decrease in yellowness of both LL and PM from *Bos taurus* cattle during storage.

#### Color stability (R630/580)

The LL steaks demonstrated greater (p<0.05) surface color stability (R630/580) on days 5 and 9 (Figure 2), whereas LL and PM exhibited similar (p> 0.05) R630/580 values on day 0. The R630/580 estimates surface discoloration; greater ratio indicates lower surface metmyoglobin accumulation (brownish discoloration) and consequently greater redness and color stability [24]. The observed differences in R630/580 values between LL and PM could be attributed to differences on muscle fiber composition [7] and muscle metabolism [8]. The PM is mainly composed of type I fiber [27], which exhibits greater oxidative metabolism and oxygen consumption [10] than glycolytic muscles, such as LL [27]. This in turn favors accumulation of metmyoglobin on surface and meat discoloration, resulting in a decrease in R630/580 values [10,16,21]. In addition, the differences in R630/580 could also be influenced by the differences in meat pH [29]. Lower ultimate meat pH (as observed in LL) decreases mitochondria respiration, thereby increasing the availability of oxygen to bind to Mb, and consequently increases surface oxymyoglobin levels and R630/580 [29]. Supporting our results, Joseph et al [14] documented greater R630/580 in LL steaks than in their PM counterparts from *Bos taurus* cattle on days 5 and 9 of retail display. In partial agreement, Canto et al [16] reported greater R630/580 values on LL steaks than in their PM counterparts from grass-fed *Bos indicus* cattle throughout 9 days of refrigerated storage. Our results differ with those of Canto et al [16] probably due to the variations in vitamin E in diet, which exerts a protective against the lipid oxidation enhancing the color stability of meat [31]. Moreover, Wu et al [21] reported a greater metmyoglobin accumulation in PM steaks than in their LL counterparts from Chinese Luxi cattle throughout 15 days of storage.

The PM steaks demonstrated a decrease (p<0.05) on R630/580 from day 5 to 9 (Figure 2), whereas in LL steaks the R630/580 values remained unchanged (p>0.05) throughout the storage. The decline in R630/580 may associated with the differences in meat pH [21]. An increase of meat pH during storage (as observed in LL and PM) enhances mitochondrial oxygen consumption leading to a decrease in surface oxymyoglobin [32] and a decline in R630/580 [29]. In partial agreement, a decrease on surface color stability of both LL and PM muscles has been previously reported in meat from *Bos taurus* [14], grass-fed *Bos indicus* [16] and Chinese [21] cattle during storage.

### Metmyoglobin reducing activity

**Figure 3:**
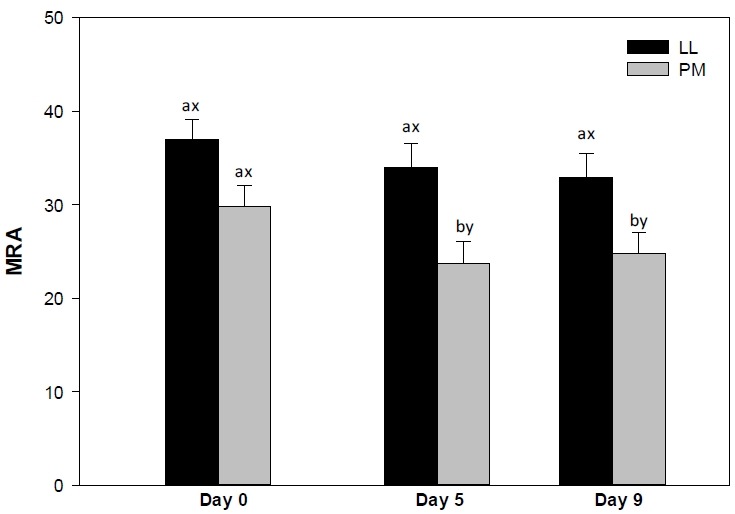
Metmyoglobin reducing activity (MRA) of *longissimus lumborum* (LL) and *psoas major* (PM) steaks from grain-fed Nellore (*Bos indicus*) bulls during aerobic storage at 4°C for 9 days. Standard error bars are indicated. ^a–b^ Means within a muscle without common superscripts are different (p<0.05). ^x–y^ Means within a day of storage without common superscripts are different (p<0.05).

The LL steaks exhibited greater (p<0.05) MRA than their PM counterparts on days 5 and 9 (Figure 3), whereas both muscles exhibited similar (p>0.05) values on day 0. The differences in MRA between LL and PM could be attributed to differences in nicotinamide adenine dinucleotide (NADH) content in the muscles [11]. NADH is the main component involved in metmyoglobin (MMb) reduction [6,10] and is regenerated by lactate dehydrogenase (LDH) in postmortem skeletal muscles [11]. Glycolytic muscles such as LL had greater LDH activity than oxidative muscles such as PM [11]. This in turn improves the regeneration of NADH and favors the reduction of MMb [21], ultimately contributing to the greater MRA in LL steaks than in PM ones [11,16,21].

In partial agreement, Abraham et al [15] reported greater MRA in LL than in PM steaks from *Bos taurus* cattle during 7 days of retail display. In addition, previous investigations reported a greater MRA in LL than in PM counterparts from *Bos taurus* cattle [14] and grass-fed *Bos indicus* animals [16]. McKenna et al [6] evaluated the MRA in 19 muscles from *Bos taurus* cattle and reported that the LL steaks exhibited greater MRA than their PM counterparts. Moreover, Wu et al [21] reported a greater MRA in LL than in PM steaks from Chinese cattle on days 0 and 5. On contrary, Jeong et al [7] documented no differences in MRA of *longissimus thoracis et lumborum* and PM steaks from Hanwoo cattle. The PM steaks exhibited a decrease (p<0.05) in MRA from day 5 to 9 of storage, whereas LL steaks demonstrated no changes (p>0.05) in the MRA. The steady MRA in LL steaks could be attributed to its high concentration of NADH [11]. Glycolytic muscles, such as LL, exhibit high LDH activity, which thereby favors the replenishment of NADH, increasing the MRA [11]. Conversely, the decrease in MRA of PM could be attributed to depletion of NADH during storage, which in turn enhances the surface MMb accumulation and meat discoloration [21]. In partial agreement, previous investigations reported a decrease in MRA of both LL and PM muscles from *Bos taurus* animals [14,15], Chinese cattle [21], and grass-fed *Bos indicus* animals [16] during storage.

### Lipid oxidation (thiobarbituric acid reactive substances)

The PM steaks demonstrated greater (p<0.05) TBARS than LL counterparts on days 5 and 9 (Table 1), whereas the lipid oxidation was not detected in both muscles on day 0. The observed variations in TBARS between LL and PM steaks could be attributed to the differences in muscle fiber characteristics [21]. Muscles composed of muscle fiber type I such as PM [27] exhibit greater content of mitochondria and oxidative metabolism than muscles composed of fiber IIB, such as LL [27]. In addition, muscles composed of fiber type I, contain high amount of lipid, utilized as energy source, and Mb content than the muscles composed of fibers type IIB [7,8,13]. These muscle fiber differences favors lipid oxidation and subsequently the increase of TBARS, at a greater degree in PM than in LL steaks [13,19]. In addition, Joseph et al [14] reported a low level of antioxidant proteins in PM than in LL muscles, which in turn could be correlated with an increase of susceptibility to lipid oxidation and subsequent decrease on color stability.

In agreement with our findings, Joseph et al [14] documented greater TBARS in PM than in LL steaks from *Bos taurus* cattle on day 9 of storage. In addition, Canto et al [16] reported greater TBARS in PM than in LL steaks from grass-fed *Bos indicus* cattle on days 3 and 9 of storage. Wu et al [21] documented greater TBARS in PM than in LL steaks from Chinese cattle on days 5 and 15 of storage. In addition, McKenna et al [6] reported greater TBARS in PM than in LL muscles from *Bos taurus* cattle during retail display. Moreover, Joseph et al [14] reported greater TBARS in PM than in LL steaks from *Bos taurus* cattle on day 9 of retail display. In partial agreement, Ma et al [13] documented similar lipid oxidation (TBARS) in LL and in PM steaks from crossbred Angus×Simmental cattle on day 0 of storage, whereas a greater TBARS was observed in PM than in LL steaks on days 9 and 16 of aging. In contrast, Jeong et al [7] did not observe differences in TBARS between LL and PM muscles from Hanwoo cattle during storage.

During storage, both LL and PM exhibited and increase (p<0.05) on lipid oxidation (Table 1). The observed increase on lipid oxidation could be related to a decrease of redox capacity of meat and subsequently generation of free radicals during storage [33]. These radicals trigger the chain reaction of lipid oxidation leading to an increase in TBARS [33]. An increase on TBARS in LL and PM muscles has been previously reported in *Bos taurus* [6,14] Chinese cattle [21], and grass-fed *Bos indicus* cattle [16].

## CONCLUSION

The findings of the present study indicate that the muscle source influenced on color stability of beef from grain-fed *Bos indicus* animals. The LL steaks demonstrated greater color, oxidative stabilities than PM steaks, which could be attributed to the differences of muscle biochemistry. These results highlighted the necessity of muscle-specific injection enhancement, and packaging strategies [34] to improve the color stability of beef from grain-fed *Bos indicus* cattle.

## Figures and Tables

**Table 1:** Meat pH, instrumental color and lipid oxidation of LL and PM steaks from grain-fed Nellore (*Bos indicus*) bulls during aerobic storage at 4°C for 9 days

Parameter	Muscle	Days of storage		

		0	5	9
Meat pH	LL	5.55±0.03^b^^y^	5.57±0.03^b^^y^	5.62±0.03^a^^y^
	PM	5.67±0.02^b^^x^	5.68±0.03^b^^x^	5.72±0.02^a^^x^
L* value	LL	39.49±0.82^a^^x^	38.55±0.64^a^^x^	38.00±0.62^a^^x^
	PM	39.52±0.27^a^^x^	38.07±0.31^b^^x^	37.40±0.45^b^^x^
b* value	LL	14.17±0.98^a^^x^	15.98±0.81^a^^x^	15.83±0.91^a^^x^
	PM	14.57±0.55^a^^x^	14.78±0.42^a^^x^	14.15±0.51^a^^x^
Lipid oxidation1)	LL	ND	0.012±0.00^b^^y^	0.015±0.00^a^^y^
	PM	ND	0.016±0.00^b^^x^	0.028±0.00^a^^x^

Results expressed as average±standard error of the mean.

LL, *longissimus lumborum*; PM, *psoas major*; ND, not detected.

1) Result expressed as absorbance at 532 nm.

a–b Means without common superscripts in a row are different (p<0.05).

x–y Means without common superscripts in a column within a parameter are different (p<0.05).
